# Genetic diversity in natural populations of noble crayfish (*Astacus astacus* L.) in north-western Poland on the basis of combined SSR and AFLP data

**DOI:** 10.7717/peerj.7301

**Published:** 2019-07-29

**Authors:** Remigiusz Panicz, Łukasz Napora-Rutkowski, Sławomir Keszka, Lidia Skuza, Magdalena Szenejko, Przemysław Śmietana

**Affiliations:** 1 Department of Meat Technology, Faculty of Food Sciences and Fisheries, West Pomeranian University of Technology Szczecin, Szczecin, Poland; 2 Polish Academy of Sciences, Institute of Ichthyobiology and Aquaculture in Golysz, Chybie, Poland; 3 Department of Aquaculture, Faculty of Food Sciences and Fisheries, West Pomeranian University of Technology Szczecin, Szczecin, Poland; 4 Department of Molecular Biology and Cytology, Institute for Research on Biodiversity, Faculty of Biology, University of Szczecin, Szczecin, Poland; 5 Molecular Biology and Biotechnology Center, Faculty of Biology, University of Szczecin, Szczecin, Poland; 6 Department of Ecology and Environmental Protection, Institute for Research on Biodiversity, Faculty of Biology, University of Szczecin, Szczecin, Poland

**Keywords:** Conservation genetics, Microsatellite marker, Restoration, Genetic differentiation, Species extinction

## Abstract

**Background:**

Conservation of noble crayfish (*Astacus astacus*) populations is becoming particularly important since the number of individuals is rapidly declining across the distribution range of the species in Europe. Five crayfish populations in northwestern Poland have been constantly monitored for two decades. However, the genetic structure of these populations has not been analysed, although this information is important to devise effective conservation strategies.

**Methods:**

Noble crayfish were collected in the autumn of 2014 by scuba diving in Lakes Graniczne, Babinki, Biwakowe, Sęki and Kwisno, all of which are situated in the Bytów Lakeland of northwestern Poland. Genetic diversity of the five populations was assessed based on allele variability in nine SSR regions and six AFLP primer combinations.

**Results:**

Microsatellite results analysed with AMOVA showed that the diversity between populations corresponds to 18% of total variability, which was confirmed by similar results obtained using AFLP. Additionally, significant genetic diversity was revealed by high average F_ST_ values. All of the studied crayfish populations significantly deviated from the expected Hardy–Weinberg genetic equilibrium and were characterised by negative values of inbreeding coefficient (F_IS_).

**Discussion:**

The invariably negative inbreeding coefficients (F_IS_) suggest a low number of mating individuals, a possible consequence of the phenomenon known as genetic bottleneck. However, additional comprehensive analyses are needed to assess the genetic structure, origin and vulnerability of the remaining populations of noble crayfish in the Bytów Lakeland of northwestern Poland, which have high conservation value and are particularly important as a live genetic bank for breeding and restitution purposes.

## Introduction

The region of Pomerania in northwestern Poland is of unique importance for crayfish monitoring. This is due to the fact that biotically driven changes in the structure of noble crayfish (*Astacus astacus* L.) populations reflect those occurring in the native range of the species and have proved to be especially dynamic over the past 120 years. It is well accepted that noble crayfish populations are highly vulnerable to habitat loss, to alien species competing for food and space, and particularly to pathogens such as the water mould *Aphanomyces astaci* as the causative agent of the fatal crayfish plague (Śmietana, 2013). The Polish Red Data Book of Animals—Invertebrates has classified noble crayfish as ‘vulnerable’ (VU) since the mid-20th century (Krzywosz & Śmietana, 2005). However, active protection plans have failed to redress this situation (Śmietana, Krzywosz & Struzyński, 2004); the number of remaining populations has further decreased, especially in standing water bodies, pushing noble crayfish species in Poland to the brink of extinction. Pomerania is the last region in Poland where populations persist, particularly in the Bytów Lakeland (Schulz, Śmietana & Schulz, 2006). However, even here the number of sites where crayfish exist has dramatically decreased in recent years, leading to the prediction that the species will go completely extinct in Poland within 10 years (Śmietana, 2013).

Reasons to protect crayfish populations from extinction not only include the conservation of native fauna, but also the need to obtain restocking material for the restoration of other populations. Such meaures have the greatest potential of success in water bodies that meet the habitat requirements of crayfish and enjoy protection from threats, such as the introduction of spiny-cheek crayfish, *Faxonius limosus*, a potent carrier of *A. astaci* (Śmietana, 2013). In other European regions, such actions have reduced the decline of crayfish populations by 40–50% (Edsman et al., 2010). However, the currently low numbers of the remaining Polish populations prohibit the safe use of caught specimens without imposing a risk on source populations. Therefore, modern methods of active protection are required that enabe the production of crayfish offspring in semi-artificial aquaculture systems. A disadvantage of this approach is, however, that the restocked populations tend to be genetically rather homogenous, due to limited size of crayfish broodstock.

The Pomeranian landscape formed by glacial till of terminal moraines is characterized by notable heterogeneity, which is one of the factors favouring high biodiversity (Krebs, 1994). Therefore, populations of freshwater species inhabiting water bodies located even in close proximity could show considerable genetic diversity. This calls for comprehensive genetic tests to characterise and protect noble crayfish populations in this region. Moreover, the Bytów Lakeland host noble crayfish populations situated at the crossroads of crayfish expansions in the past. Data from genetic studies in this region would thus provide valuable information on crayfish genotype distribution and diversity relevant to crayfish management.

Microsatellites, which are also known as simple sequence repeats (SSR), are one of the most informative genetic markers. SSR are highly polymorphic, abundant and evenly distributed throughout genomes (Schlötterer, 2004). This feature makes them a popular marker to analyse genetic diversity (Groeneveld et al., 2010), including of noble crayfish populations (Gross et al., 2013; Kõiv et al., 2008; Kõiv et al., 2009; Schrimpf et al., 2014; Schrimpf et al., 2017). Microsatellites provide many advantages, but they also have shortcomings. In particular, genotyping is often complicated and difficult to automate due to the presence of so-called stutter bands and null alleles (Chapuis & Estoup, 2007).

Most studies on crayfish genetic diversity only used one type of genetic marker, such as allozymes (Fevolden, Taugbøl & Skurdal, 1994), ITS linked microsatellites (Edsman et al., 2002) or subunit I of the mitochondrial cytochrome oxidase (Schrimpf et al., 2011). However, a combination of different genetic markers enhances assessments of genetic variability within and among populations. Alternatives that have been used for noble crayfish populations are markers for random amplification of polymorphic DNA (RAPD) (Schulz, 2000) and inter simple sequence repeat (ISSR) polymorphism (Schulz, Śmietana & Schulz, 2004). Some drawbacks of other molecular methods might be overcome by assessing amplified fragment length polymorphism (AFLP), a PCR-based method to locate dominant markers witin multiple restriction sites scattered throughout the genome (Schlötterer, 2004). A major advanatge of AFLP is that a large fraction of the genome is easily screened, making it possible to generate results similar to calculations obtained based on co-dominant markers (Bensch & Åkesson, 2005).

The aim of the present study was to characterize noble crayfish populations in the Bytów Lakeland of northwestern Poland by using microsatellite and AFLP markers. This information was to serve as a basis for conservation measures for noble crayfish, complementing the ongoing monitoring of crayfish populations, including restituted populations, the selection of new water bodies suitable for restitution, the identification of genotypes for restocking to minimize impact on natural populations, and the improvement of restitution and breeding methods.

## Materials & Methods

**Figure 1:**
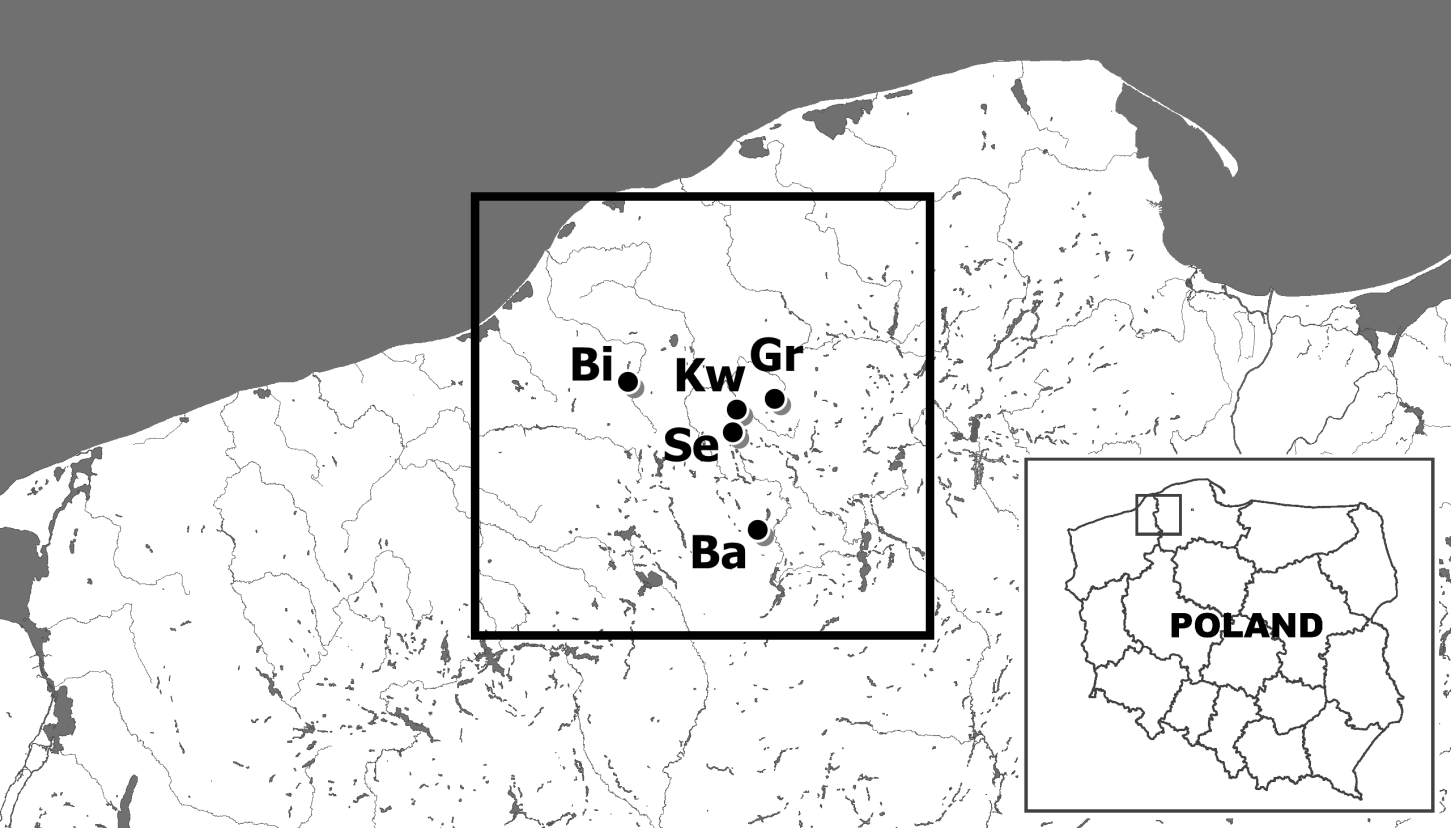
The location of sites of examined noble crayfish populations in the Bytów Lakeland. BI –Biwakowe Lake, KW –Kwisno Lake, GR –Graniczne Lake, SE –Sęki Lake, BA –Babinki Lake.

Twenty noble crayfish (*A*. *astacus*) specimens were collected from each of five lakes located in the Bytów Lakeland of northwestern Poland: Lakes Graniczne (Gr), Babinki (Ba), Biwakowe (Bi), Sęki (Se) and Kwisno (Kw). All lakes were sampled by scuba diving during the autumn of 2014 (Fig. 1). One walking leg of the fifth pair was cut off from each specimen and preserved in 75% ethanol before releasing the individuals. DNA was extracted from muscle tissue of the legs using the High Pure PCR Template Preparation Kit (Roche Diagnostics GmbH, Mannheim, Germany). Qualitative and quantitative analyses of the DNA were performed by separation on 1.5% agarose gels and spectrophotometric measurements using a NanoDrop 2000 spectrophotometer (Thermo Scientific, Wilmington, DE, USA).

### Microsatellite markers

All PCR mixtures were prepared in a final volume of 25 µL using 12.5 µL of the FastStart PCR Master kit (Roche Diagnostics), 10.5 µL water, 0.5 µL (0.2 mM) forward primer, 0.5 µL (0.2 mM) reverse primer and 1 µL DNA template. Nine SSR regions (Aas1198, Aas6, Aas3040, Aas766, Aas7, Aas3666, Aas11, Aas2, Aas8) were amplified according to the methodology described by Kobreveiv et al. (2008, 2009). A fluorescent marker dye (WellRED dye; Sigma-Aldrich, St. Louis, MO, USA), D2, D3 or D4 was attached to the 5′end of sense primers (For) from each pair. PCR products were mixed with the DNA Size Standard Kit –400 (Beckman Coulter, Brea, CA, USA) and separated by capillary electrophoresis using a CEQ 8000 automatic sequencer (Beckman Coulter).

### AFLP markers

Analysis of AFLP markers was performed according to a modified protocol by Papa et al. (2005).**** Briefly, DNA isolates (400 ng) were digested with two restriction enzymes, *Eco* RI (5U) and *Mse* I (5U) (New England Biolabs, Ipswich, MA, USA), at 37 °C for 3 h. Next, oligonucleotide adapters were ligated for 16 h at 20 °C using T4 ligase (1U) in T4 buffer (1x) (New England Biolabs). The products of this reaction were diluted 1:10 and used as a template for the following pre-amplification step. Three microliters of diluted template DNA were then added to a PCR reaction mix (total volume of 25 µL) containing Taq DNA Polymerase (1U), buffer B (1X), dNTPs (10 mM), and primers E01 and M01 at a concentration of 10 mM each. Next, one microliter of the diluted 20-fold preamplified template was added to the PCR reaction mix to a final volume of 20 µL containing 10 µL of the FastStart PCR Master Mix (Roche Diagnostics), 8.2 µL of water, 0.2 µL of 10 mM 5′ fluorescently labelled (WellRED dye) EcoRI primer and unlabelled 0.2 µL of 10 mM MseI primer, each carrying three selective nucleotides (E-AAG/M-CAA, E-AAG/M-CAC, E-AAG/M-CAG, E-AAG/M-CCT, E-AAG/M-CTC, E-AGA/M-CAG; Table 1). Pre-amplification and amplification steps were performed according to profiles described in Papa et al. (2005). PCR products were seprated on a CEQ 8000 automated capillary DNA sequencer (Beckman Coulter) along with internal DNA Size Standard 400 (Beckman Coulter).

**Table 1:** Starter sequences used in pre-amplification reactions and PCR selective amplification of the AFLP analysis.

**Starter name**	**Starter sequence (5′ → 3′)**
E01	GACTGCGTACCAATTC A
M01	GATGAGTCCTGAGTAA C
E-aag	D4- GACTGCGTACCAATTC AAG
E-aga	D2- GACTGCGTACCAATTC AGA
M-ctc	GATGAGTCCTGAGTAA CTC
M-ctt	GATGAGTCCTGAGTAA CTT
M-cac	GATGAGTCCTGAGTAA CAC
M-cag	GATGAGTCCTGAGTAA CAG
M-ctg	GATGAGTCCTGAGTAA CTG
M-cct	GATGAGTCCTGAGTAA CCT
M-caa	GATGAGTCCTGAGTAA CAA

### Microsatellite data analysis

Microsatellite Analyzer (MSA) software ver. 4.05 (Dieringer & Schlötterer, 2003) was used to calculate the number of private alleles (Apr), *F*_*IS*_ and pairwise *F*_*ST*_ values (Weir & Cockerham, 1984), observed and expected heterozygocity (H_*O*_ and H_*E*_), allelic richness (A_*R*_), and Nei’s distance D_*A*_ (Nei, Tajima & Tateno, 1983). The same genetic distance (D_*A*_) matrix was used to construct an UPGMA dendrogram and to test population grouping by bootstrapping analysis (1,000 permutations) applying the PHYLIP software package ver. 3.695 (Felsenstein, 1989). Tests for deviation from Hardy–Weinberg equilibrium (HWE) across all loci for each population were computed by GENEPOP 4.2 (Raymond & Rousset, 1995; Rousset, 2008), using Fisher’s exact test and the Markov chain algorithm to calculate *p*-values (Guo & Thompson, 1992). Sequential Bonferroni adjustments (Rice, 1989) were applied to correct for the effect of multiple tests. The number of private alleles relative to other populations from this study and principal coordinate analysis (PCoA) based on pair-wise *F*_*ST*_ values were calculated using the GENALEX 6.41 package (Peakall & Smouse, 2012). Hierarchical analysis of molecular variance (AMOVA) was computed using ARLEQUIN ver. 3.1 (Excoffier, Laval & Schneider, 2005). To investigate differences in genetic structure between populations, the approach implemented in STRUCTURE 2.3.3 (Pritchard, Stephens & Donnelly, 2000) was used. Specific conditions of the calculations included an admixture model and correlated allele frequency, for *K* = 1 to 7, 20 independent runs for each K value with 5 × 10^5^ Markov chain Monte Carlo iterations after a burning period of 5 × 10^5^ repetitions. The evaluation of the best K genetic cluster was based on ΔK following the Evanno method (Evanno, Regnaut & Goudet, 2005) using the STRUCTURE HARVESTER v 0.6.91 application (Earl & Von Holdt, 2012). The population assignment test was performed using GeneClass2 (Piry et al., 2004) software with a frequencies-based method by Paetkau et al. (1997).

### AFLP data analysis

Fragment data were transferred to a binary (1/0) data matrix using the software GenomeLab (Beckman Coulter). The data matrix was then imported to FAMD ver. 1.25 (Fingerprint Analysis with Missing Data) (Schlüter & Harris, 2006) to estimate the number of polymorphic markers, percentage of polymorphic markers in every population, hierarchical partition of genetic diversity evaluated by analysis of molecular variance (AMOVA; Excoffier, Smouse & Quattro, 1992), Cavalli-Sforza & Edwards’ chord distance (1967) among pairs of populations to construct an UPGMA majority rule consensus bootstrap (×1, 000) population tree and perform a PCoA.

## Results

### SSR marker analysis

Among the 9 studied microsatellite loci, a total of 59 alleles were detected across the 5 crayfish populations that were analysed. The mean number of alleles per locus was 6.55 ranging from 4 alleles for loci Aas3040 and Aas11 to 10 for locus Aas3666 (Table 2). The overall average observed heterozygosity (H_*O*_) was 0.470 and ranged from 0.05 for Aas2 to 1.00 for Aas766 and Aas7. The average expected heterozygosity (H_*E*_) per locus was 0.380 and ranged from 0.109 for Aas2 to 0.655 for Aas1198. Twenty population-specific alleles (private alleles) were identified (Table 2).

### Genetic diversity within populations

The mean number of alleles expressed here as mean A_*R*_, rarefied to a sample size of 20 individuals, reached the highest value of 4.44 in the Gr population and a slightly lower value of 4.33 in the Ba population; the lowest value of 2.44 was observed for the Bi population (Table 3). The observed heterozygosity (H_*O*_) ranged from 0.422 for the Bi and Se populations to 0.506 for the Gr and 0.511 for the Ba population, whereas the expected heterozygosity (H_*E*_) ranged from 0.293 for the Se to 0.448 for the Gr population (Table 3). In all populations, most of the analysed loci significantly deviated from HWE. Significant to highly significant deviations from HWE were observed in 33 out of 45 cases (9 loci × 5 populations). There was a large excess of heterozygotes in all analysed populations across the 9 loci, which was revealed by strongly negative values of averaged *F*_*IS*_, which varied from −0.420 for the Se population to −0.102 for the Gr population (Table 3). The highest number of 10 population-specific alleles was observed in Gr. Five such alleles were observed in Ba, 3 in Bi and 2 in Kw, whereas the Se populations had no private alleles (Table 3). In population Bi, 3 of the analysed SSR loci were monomorphic, 2 monomorphic loci were observed in the Se population and 1 in the populations of Ba and Kw.

**Table 2:** Characteristics of the studied microsatellite loci.

**Locus**	**Allele size range (bp)**	**A**	**H_*O*_**	**H_*E*_**	**Apr**
Aas1198	173–193	9	0.990	0.655	2
Aas6	144–180	5	0.210	0.199	2
Aas3040	239–245	4	0.330	0.381	1
Aas766	285–297	7	1.000	0.620	1
Aas7	256–282	9	1.000	0.649	3
Aas3666	227–255	10	0.300	0.336	4
Aas11	177–197	4	0.150	0.206	1
Aas2	155–177	6	0.050	0.109	4
Aas8	183–207	5	0.200	0.264	2
**Average across populations**	****	**6.55**	**0.470**	**0.380**	****

**Notes.**

A total number of observed alleles H_*O*_ average observed heterozygosity H_*E*_ average expected heterozygosity Apr number of private alleles

**Table 3:** Genetic variability within the studied noble crayfish populations using 9 microsatellite markers.

**Population code**	**A_*R*_**	**H_*O*_**	**H_*E*_**	**P_*HW*_**	**F_*IS*_**	**Apr**	**Monomorphic loci**	**Assignment test**
Gr	4.44	0.506	0.460	^***^	−0.102	10	–	65%
Ba	4.33	0.511	0.453	^***^	−0.132	5	Aas6	50%
Bi	2.44	0.422	0.303	^***^	−0.410	3	Aas6, Aas2, Aas8	95%
Se	2.77	0.422	0.301	^***^	−0.420	0	Aas11, Aas2	85%
Kw	2.77	0.489	0.383	^***^	−0.285	2	Aas2	95%

**Notes.**

*** *p* < 0.001.

significance values A_*R*_ mean values of allelic richness H_*O*_ average observed heterozygosity H_*E*_ average expected heterozygosity P_*HW*_ probability of deviations from expected Hardy–Weinberg proportions after sequential Bonferroni adjustments F_*IS*_ inbreeding coefficient Apr number of private alleles Assignment test percent of correctly assignment noble crayfish to their population of origin

### Genetic relationships and variation among populations

**Figure 2:**
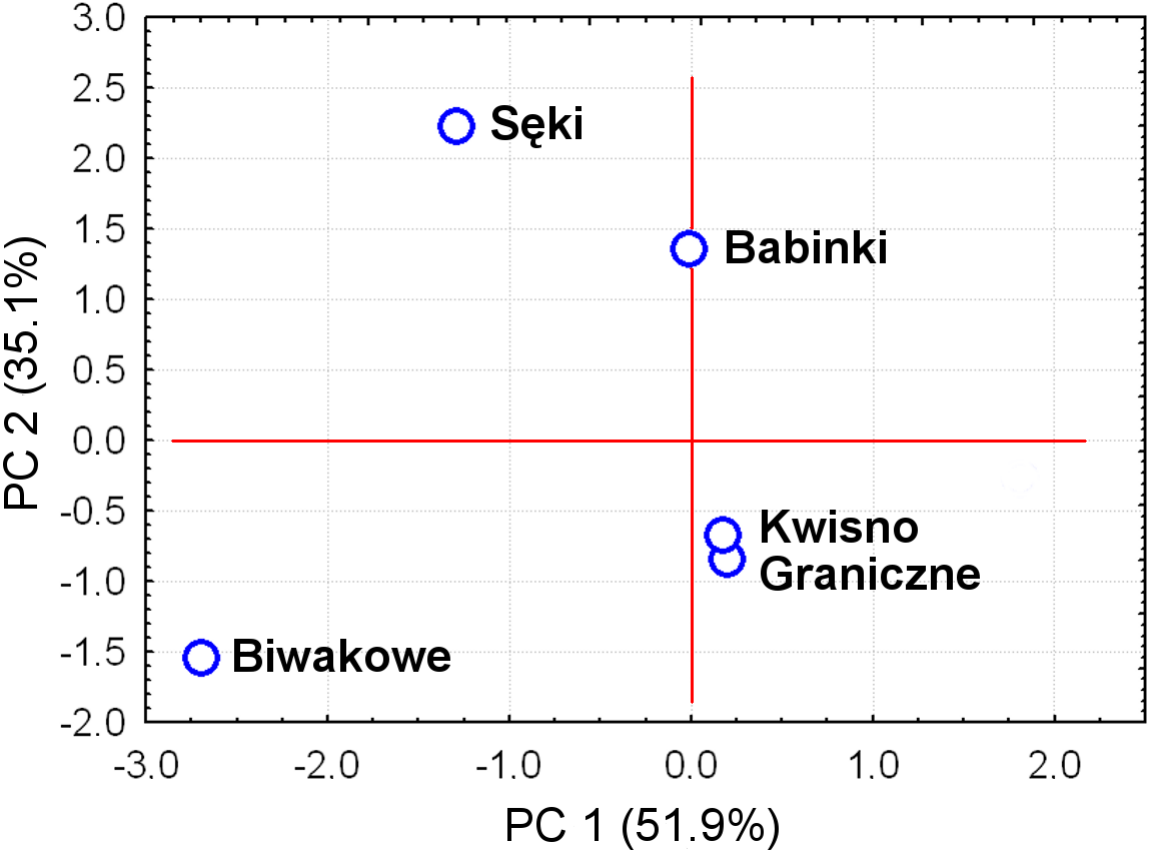
Principal Coordinates Analysis (PCoA) of the Fst values between studied populations of the noble crayfish. PCoA of the Fst values calculated between crayfish populations (marked as blue circles) differentiates Se and Ba from Gr and Kw, and Bi.

**Figure 3:**
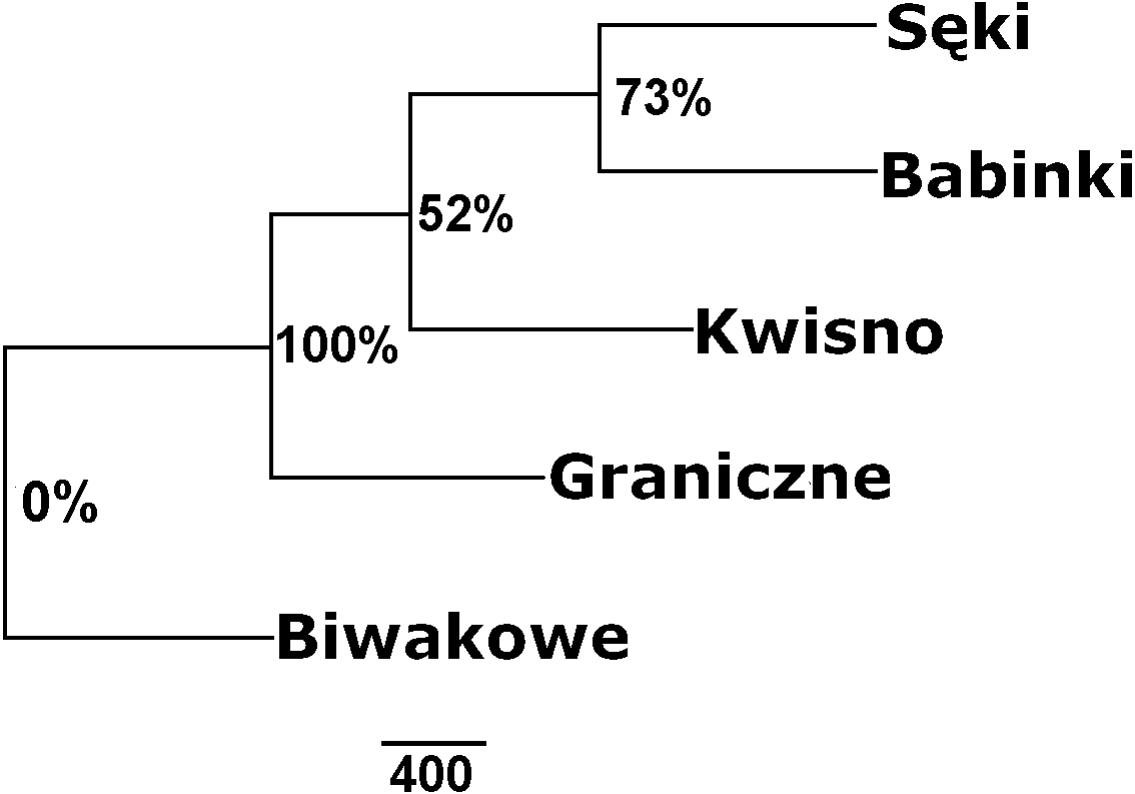
Unrooted UPGMA clustering of noble crayfish populations based on SSR loci and Nei’s chord distance (1983) (Da) among pairs of populations. Numbers indicate clades bootstrap support in 1,000 replicates.

**Figure 4:**
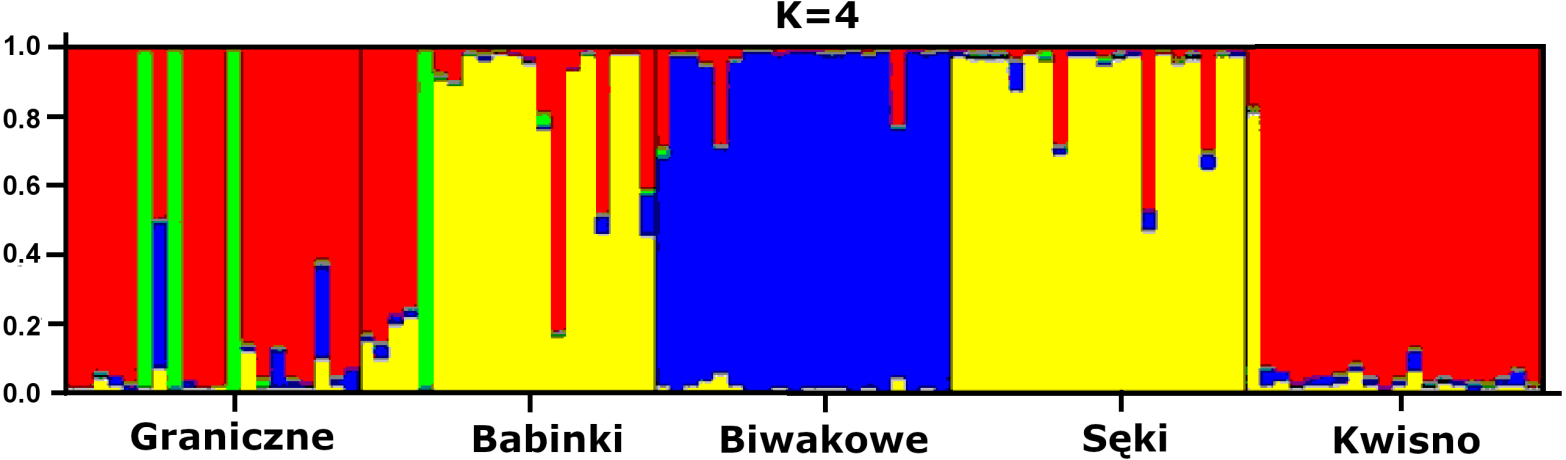
Results of the hierarchical STRUCTURE analysis of the five noble crayfish populations under analysis. K refers to the number of inferred genetic clusters. The black lines separate individuals of different populations.

The average genetic differentiation (*F*_*ST*_) among the five analysed crayfish**** populations was relatively high (*F*_*ST*_ = 0.183). Pairwise estimates of *F*_*ST*_ ranged from 0.038 (between the Gr and Kw populations) to 0.348 (between the Bi and Se populations) (Table 4). PCoA of the F_*ST*_ values between the studied populations differentiates the pair of the Se and Ba populations from the pair of the Gr and Kw populations. The Bi population was distinct from all other investigated populations (Fig. 2). Global AMOVA results, as a weighted average over 9 loci, attributed 18.3% of the total variation to variability between the analysed populations and most of it (81.7%) was due to variance within populations. The UPGMA dendrogram constructed on the basis of the Da population distances separated the Gr population into a distinct clade with a very high bootstrap value (100%; Fig. 3). The Se and Ba population pairs constructed separate groups with 72.8% bootstrap support (Fig. 3). The best ΔK value in the Bayesian clustering analysis using the five populations of noble crayfish was obtained for *K* = 4 (Fig. 4). The population pair of Gr and Kw was grouped into one cluster, populations Ba and Se were grouped into another cluster, and a third separate cluster was created for the Bi population (Fig. 4).

**Table 4:** Pairwise F_*ST*_ values between studied populations of the noble crayfish (below diagonal), and significance values of allelic differentiation for each population pair (above diagonal) after sequential Bonferroni adjustments.

	**Gr**	**Ba**	**Bi**	**Se**	**Kw**
**Gr**	0.0000	^*^	^*^	^*^	^*^
**Ba**	0.1167	0.0000	^*^	^*^	^*^
**Bi**	0.1852	0.2806	0.0000	^*^	^*^
**Se**	0.2298	0.0573	0.3483	0.0000	^*^
**Kw**	0.0382	0.1265	0.2130	0.2107	0.0000

**Notes.**

* *p* < 0.05—significance values of allelic differentiation.

### AFLP marker analysis

**Figure 5:**
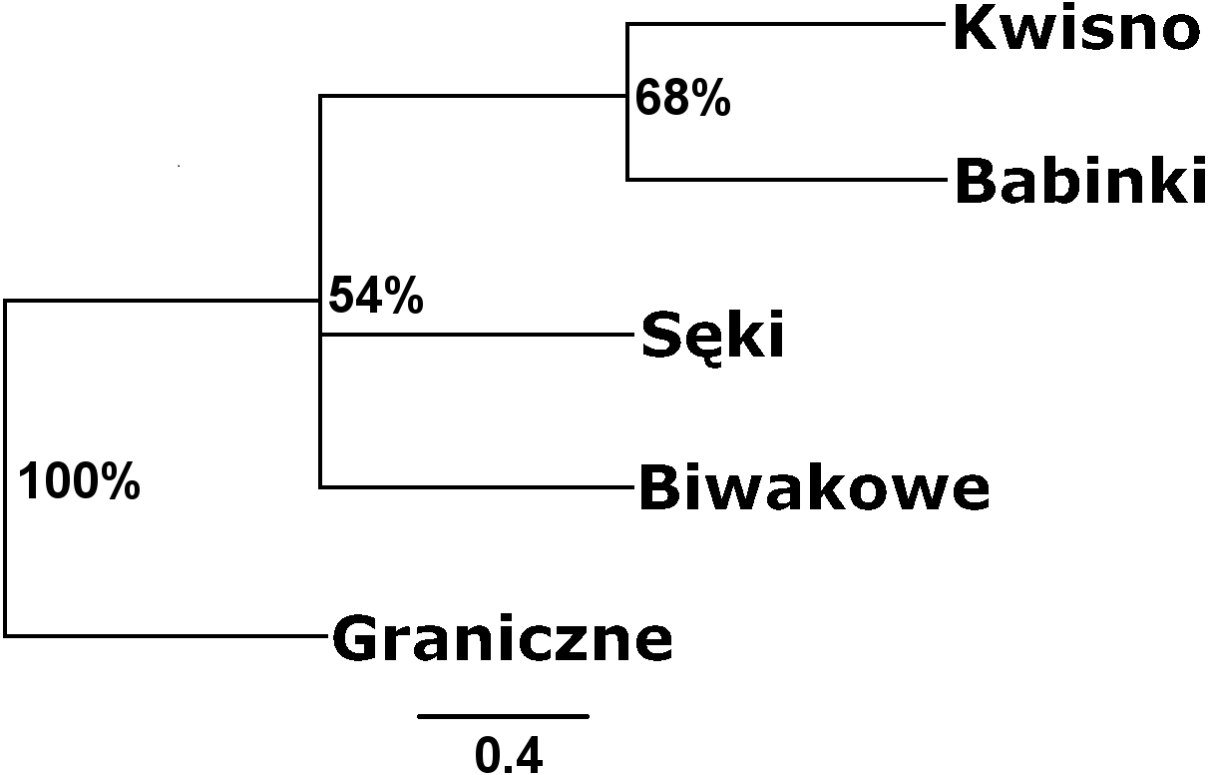
Unrooted UPGMA clustering of noble crayfish populations based on AFLP loci and Cavalli-Sforza & Edwards (1967) distances among pairs of populations. Numbers indicate clades bootstrap support above 50% in 1,000 replicates.

**Figure 6:**
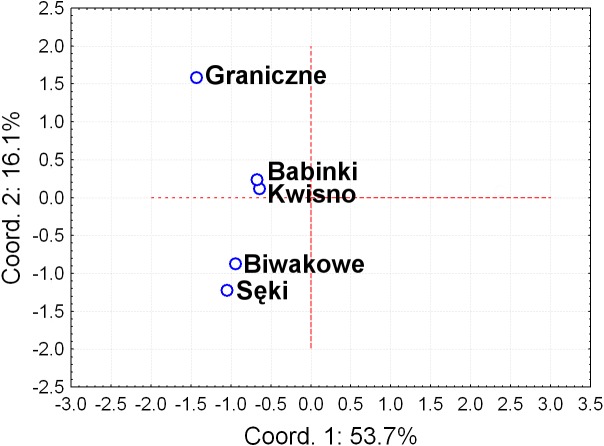
Principal Coordinates Analysis (PCoA) of the based on AFLP Cavalli-Sforza & Edwards (1967) distances between analysed populations of the noble crayfish. Graph represents distances calculated between five crayfish populations (marked as blue circles).

Seven pairs of PCR primers in selective amplification generated 290 AFLP polymorphic markers in total. DNA fragments with lengths ranging from 60 to 450 bp were analysed. The AFLP profiles of individuals of the analysed crayfish population comprised an average of 172 loci, where individuals from the Se population were characterized by profiles with the lowest average number of AFLP markers, amounting to 158. Profiles with the largest average number of AFLP markers (182.1) were observed in the Gr population (Table 5). The lowest number of polymorphic AFLP loci was observed in the Bi population (69.0%) and the largest in the Kw population (77.9%) (Table 5). AMOVA of the genetic diversity of AFLP markers of the analysed crayfish populations showed that 16.2% of the genetic diversity occurred between populations and the larger part (84%) within populations. The UPGMA dendrogram constructed on the basis of the Cavalli-Sforza & Edwards (1967) population distance separated the Gr population from all others, which wa supported by a 100% bootstrap value (Fig. 5). A population pair with 68% bootstrap support was found between populations Kw and Ba and 54% between Kw/Ba and Se/Bi populations (Fig. 5). A PCoA based on Cavalli-Sforza and Edwards’ (1967) population distance explicitly differentiated noble crayfish populations of Lake Graniczne, Sęki and Biwakowe (Fig. 6).

**Table 5:** Descriptive statistics for each of the 5 lake populations examined using the AFLP markers.

**Population code**	**N**	**Polymorphic loci (%)**	**Marker presences per individual**
Gr	9	77	182
Ba	9	70	184
Bi	9	69	164
Se	8	72	158
Kw	9	78	173

**Notes.**

N sample size

## Discussion

The analysis of genetic diversity among and within natural populations of noble crayfish in northwestern Poland was conducted using two types of genetic markers, SSR and AFLP. Both revealed significant genetic differentiation in the five analysed populations. One of the most important factors influencing genetic diversity of the crayfish populations from the Bytów Lakeland is a diverse topography shaped by the action of glaciers (Lindner & Marks, 2008). Lakes located in close proximity (three km) but separated by a deep valley (e.g., Kwisno and Sęki) host crayfish populations regarded as genetically distinct (*F*_*ST*_ = 0.211; Table 4). In comparison, large interconnected water bodies in Finland enable uninterrupted crayfish movement which led to genetic homogenization and an overall loss of genetic diversity.

Generally open lakes facilitate movement and hence new combinations of alleles in the offspring; unless there is selection pressure on populations towards homo- or heterozygosity, the genetic balance is thus maintained (Alaranta et al., 2006). The two adjacent lakes in our study, Kw and Se, also belong to different catchments, that of the Wieprza River flowing north and entering the Baltic Sea and that of the Brda River flowing south as a tributary to the Vistula River. Kwisno is an example of a water body which might have lost its connection to the Wieprza River as a result of a lowered water level after the last glaciation (Lindner & Marks, 2008). The crayfish population in this isolated lake is characterised by an *F*_*IS*_ coefficient (heterozygote excess) of –0.285, a low allelic richness value (2.77) and a statistically significant deviation from the HWE in the genotype distribution (Table 3). All indices suggest that the crayfish population from Lake Kwisno has a small reproductive size with only few breeders likely to contribute to the next generation. Reasons for heterozygote excess in crayfish populations remain poorly understood, although several mechanisms potentially account for negative *F*_*IS*_ coefficients. These include a low number of mating individuals, selection pressure towards heterozygotic individuals, and asexual reproduction (Stoeckel et al., 2006). Gross et al. (2013) reported negative *F*_*IS*_ values for several crayfish populations from Sweden (Ljungan, Måssjön, Skiringen), Finland (Linkullasjön, Mikkolanjärvi) and Estonia (Laugi). In contrast to the populations studied here, all mean *F*_*IS*_ values calculated for populations in those countries were positive (Table 3).

Except for evidence obtained from topography analysis, genetic results confirmed that populations from Kwisno and Graniczne Lakes had the lowest level of genetic differentiation (*F*_*ST*_ = 0.04). Lake Graniczne has a permanent connection to the Pokrzywna River, a left-bank tributary of the Wieprza River. Therefore, it is conceivable that populations could move between the two lakes in the past and thus interbreed. However, over the years, Lake Kwisno and the Wieprza River became disconnected and a subpopulation could emerge as a result of a founder effect, a mechanism that has been widely described also for other species (Bernatchez & Wilson, 1998). *F*_*ST*_ values support the notion that Kwisno emerged as a part of the Lake Graniczne-Wieprza River population (Figs. 2 and 4, Table 4), whereas the reduction in genetic diversity revealed by values of allelic richness, *F*_*IS*_ and Apr are in line with the assumption that a founder event occurred in the past (Table 3). Furthermore, interviews with local inhabitants suggest that the population of Lake Kwisno experienced extensive illegal exploitation, which could have further reduced diversity of the genetic pool (Śmietana, 2013). A similar situation was identified for Lakes Sęki and Babinki in the Brda River catchment. Lake Sęki, similar to Lake Kwisno, is an isolated body of water. The crayfish population inhabiting this lake is characterised by a negative *F*_*IS*_ coefficient of −0.420 (Table 3). An analysis of the diverse topography of the Bytów Lakeland (Śmietana, 2013) juxtaposed with the present genetic results reveal that in the past the population from Lake Sęki lost the connection with the populations in the Brda River catchment, including the population in Lake Babinki. The crayfish from Lake Babinki belong to a large and non-isolated population that shows no signs of disruptive circumstances in the past that could have changed the distribution of genotypes and allele frequencies (Table 3). Instead, a high degree of similarity between the populations of the two lakes (*F*_*ST*_ = 0.057) suggests common ancestors, further supporting the above conclusions derived from the use of other methods (Figs. 2–4).

Among all results of the present study, those on Lake Biwakowe are the most interesting. The crayfish population of this lake is characterised by the highest diversity of all populations investigated here (Figs. 2, 4, 5 and 6). In contrast, genetic variability of the populations in Lakes Seki and Kwisno was considerably reduced, as shown by a low *F*_*IS*_ value of −0.410 (Table 3). Differences in genetic diversity (*F*_*ST*_) between the investigated populations was greatest between Lake Biwakowe and the lakes of the Brda catchment. This outcome is most likely the result of geographic isolation, which has probably existed since the Bytów Lakeland was formed when the glacier receded after the last glaciation (Willisa & Van Andelb, 2004; Marks, 2012). Significant differences between populations can indeed result from landscape characteristics, as described for the genetic divergence of populations of Pacific jumping mice (*Zapus trinotatus*; Vignieri, 2005), tiger salamanders (*Ambystoma tigrinum melanostictum*; Spear et al., 2005) or long-toed salamanders (*Ambystoma macrodactulym*; Giordano, Ridenhour & Storfer, 2007). Results obtained here for noble crayfish from the Bytów Lakeland provide another such example for populations of a threatened species.

Significant genetic separation of the five investigated populations is also confirmed by the results of AMOVA on the microsatellite loci, showing that the diversity among populations corresponds to 18% and 82% of the total and intrapopulation variability, respectively. Variability at this level was also confirmed by the AFLP results. Additionally, an apparent grouping of populations was confirmed by the STRUCTURE analysis, which designated four separate clades (*K* = 4) among the five populations (Fig. 4). These results indicate genetic differentiation of the noble crayfish population in Lakes Kwisno and Graniczne from those in Lakes Babinki and Sęki. The geographically isolated population of Lake Biwakowe, which has no surface water connection with the other four lakes, proved to be genetically distinct from all others. This finding is in agreement with possible routes of crayfish movement, since Lakes Kwisno and Graniczne belong to the same catchment of the Wieprza River, whereas Lakes Babinki and Sęki are part of the Brda River catchment.

The findings of the preent study add information to the results of a large-scale survey about the diversity, recolonization routes and structure of noble crayfish populations in Europe (Gross et al., 2013). Gross et al. (2013) determined the lowest mean A_*R*_ value for Finnish (2.0) and the highest value for German (4.2) populations. The populations of the Bytów Lakeland investigated here had an intermediate value (3.3). Similarly, the average H_*O*_ or H_*E*_ values of 0.470 and 0.380, respectively, in the present study ranged between the highest and lowest average values for the German (H_*O*_ = 0.533, H_*E*_ = 0.598) and Finnish (H_*O*_ = 0.212, H_*E*_ = 0.228) populations. Strong within-country genetic differentiation of noble crayfish was observed in Estonia, probably due to limited gene fiow among populations in different catchments as well as to genetic drift (Gross et al., 2013). Notably, this level of within-region variation was restricted to Estonia, where both Baltic and Black Sea genotypes were observed (Gross et al., 2013). A similar situation might exist in Poland where populations of different catchments show strong genetic differentiation. Given its geographical position, Poland might be a region where the southern and northern genotypes identified by Gross et al. (2013) co-occur as well. This makes the area particularly interesting and important to determine the genetic structure of noble crayfish populations. The indices obtained in the present study confirm the northern direction of crayfish re-colonisation along with decreasing genetic diversity, as is commonly observed also for other species (Taberlet et al., 1998; Hewitt, 1999). However, for an unambiguous reconstruction of crayfish colonisation routes after glaciation, an analysis of peri-Baltic populations is required, based on a large number of microsatellite markers (or SNP) and in combination with mitogenome analyses.

An assessment of the size of noble crayfish populations in selected lakes of Pomerania by standard methods (i.e., capture per unit effort, CPUE) (Stuecheli, 1991; Dorn & Wojdak, 2005) showed that crayfish were most abundant in Lake Graniczne and occurred at only slightly lower densities in Lake Sęki (Śmietana, 2013). However, while fluctuations in population size were the lowest in Lakes Kwisno and Babinki, the greatest reduction in the population size was observed in Lake Sęki, a small lake which had probably been subject to extensive illegal exploitation (Śmietana, 2013). Strong exploitation pressure might also explain the considerable decrease in crayfish genetic diversity in that lake.

Research in the Bytów Lakeland over the past 20 years has shown that noble crayfish may be an indicator of aquatic habitat quality (Śmietana, 2013). Numbers have exponentially declined in the area, apparently due to habitat deterioration by eutrophication, poaching, eel stocking and the invasion of non-indigenous crayfish species carrying the crayfish plague. At present, the most favourable habitats for the species in Pomerania occur in isolated areas at higher altitude with cooler and more humid climate (Śmietana, 2013). The location of the lakes where noble crayfish were caught for the present study reflects geomorphological changes in the Bytów Lakeland since the last glaciation approximately 10,000 years ago (Marks, 2012). The area is characterized by high landscape diversity with numerous moraine uplands rising to ridges above 200 m a.s.l. and deep subglacial gullies partly filled with lakes and river valleys (Raczkowski & Sroka, 2010). Lakes in the area currently inhabited by noble crayfish are characterised by forest cover in the catchments close to 85% (Śmietana, 2013). This and the results presented here indicate that anthropogenic disturbance have a notable influence on crayfish populations in the area (Śmietana, 2013). Lake Graniczne situated at 27.9 km from the nearest human settlement had the highest allelic richness and Lake Biwakowe (5.7 km distance) had the lowest, while the three other crayfish populations in Lakes Babinki (18.8 km), Sęki (11.4 km) and Kwisno (16.8 km) were characterised by intermediate values (Table 3). Poaching, eel stocking, the crayfish plague transmitted by non-indigenous crayfish species, all of which are related to human activities, apparently had the greatest impact (Śmietana, 2013). This and the evidence presented here suggest that noble crayfish should be valued for nature conservation in Pomerania as much as the animal species listed in the Habitats Directive of the European Commission (1992).

## Conclusions

The five crayfish populations investigated in the present study have been constantly monitored for two decades. Results of these monitoring efforts indicate that conservation of the populations requires active protection measures such as restocking with offspring obtained especially through aquaculture breeding programmes of crayfish with known genetic make-up. DNA markers facilitated the genetic characterisation of selected remaining natural populations of *A. astacus* in norhwestern Poland. Given their diversity, the last populations of noble crayfish in the Bytów Lakeland analysed in the present study have great value as a live genetic bank for such breeding and restitution purposes. As the results presented here show, genetic analyses of the populations are also valuable to inform about the genetic diversity, post-glacial re-colonisation routes and population structure of noble crayfish populations in Europe. However, additional comprehensive analyses are needed to assess the genetic structure, origin and vulnerability of the remaining natural populations in norhwestern Poland.

##  Supplemental Information

10.7717/peerj.7301/supp-1 Dataset S1 Microsatellite loci for evaluating genetic diversity of 5 populations of A. astacus Raw data for microsatellites (SSR) obtained for 5 populations of noble crayfish. BI –Biwakowe Lake, KW –Kwisno Lake, GR –Graniczne Lake, SE –Sęki Lake, BA –Babinki Lake

## Additional Information and Declarations

### Data Availability

The following information was supplied regarding data availability: The raw data is available as Dataset S1.
